# Comprehensive landscape of extracellular vesicle-derived RNAs in cancer initiation, progression, metastasis and cancer immunology

**DOI:** 10.1186/s12943-020-01199-1

**Published:** 2020-06-05

**Authors:** Wei Hu, Cong Liu, Zhuo-Yue Bi, Qun Zhou, Han Zhang, Lin-Lin Li, Jian Zhang, Wei Zhu, Yang-Yi-Yan Song, Feng Zhang, Hui-Min Yang, Yong-Yi Bi, Qi-Qiang He, Gong-Jun Tan, Cheng-Cao Sun, De-Jia Li

**Affiliations:** 1 Department of Preventive Medicine, School of Health Science, Wuhan University, No.115 Donghu Road, Wuhan, Hubei 430071 People’s Republic of China; 2 Hubei Provincial Key Laboratory for Applied Toxicology (Hubei Provincial Academy for Preventive Medicine), Wuhan, Hubei 430079 People’s Republic of China; 3 Department of Clinical Laboratory, Zhuhai Hospital, Jinan University, 79 Kangning Road, Zhuhai, Guangdong 519000 People’s Republic of China; 4 Department of Biomedical Engineering, University of Houston, Houston, TX 77204 USA; 5 Department of Molecular and Cellular Oncology, The University of Texas MD Anderson Cancer Center, Houston, TX 77030 USA; 6 Population and Health Research Center, School of Health Sciences, Wuhan University, Wuhan, Hubei 430071 People’s Republic of China

**Keywords:** Extracellular vesicle, Exosome, Microvesicle, Micro RNA, Long non-coding RNA, Circular RNA, Cancer, Tumor microenvironment, Premetastatic niche

## Abstract

Extracellular vesicles (EVs), a class of heterogeneous membrane vesicles, are generally divided into exosomes and microvesicles on basis of their origination from the endosomal membrane or the plasma membrane, respectively. EV-mediated bidirectional communication among various cell types supports cancer cell growth and metastasis. EVs derived from different cell types and status have been shown to have distinct RNA profiles, comprising messenger RNAs and non-coding RNAs (ncRNAs). Recently, ncRNAs have attracted great interests in the field of EV-RNA research, and growing numbers of ncRNAs ranging from microRNAs to long ncRNAs have been investigated to reveal their specific functions and underlying mechanisms in the tumor microenvironment and premetastatic niches. Emerging evidence has indicated that EV-RNAs are essential functional cargoes in modulating hallmarks of cancers and in reciprocal crosstalk within tumor cells and between tumor and stromal cells over short and long distance, thereby regulating the initiation, development and progression of cancers. In this review, we discuss current findings regarding EV biogenesis, release and interaction with target cells as well as EV-RNA sorting, and highlight biological roles and molecular mechanisms of EV-ncRNAs in cancer biology.

## Background

**Fig. 1:**
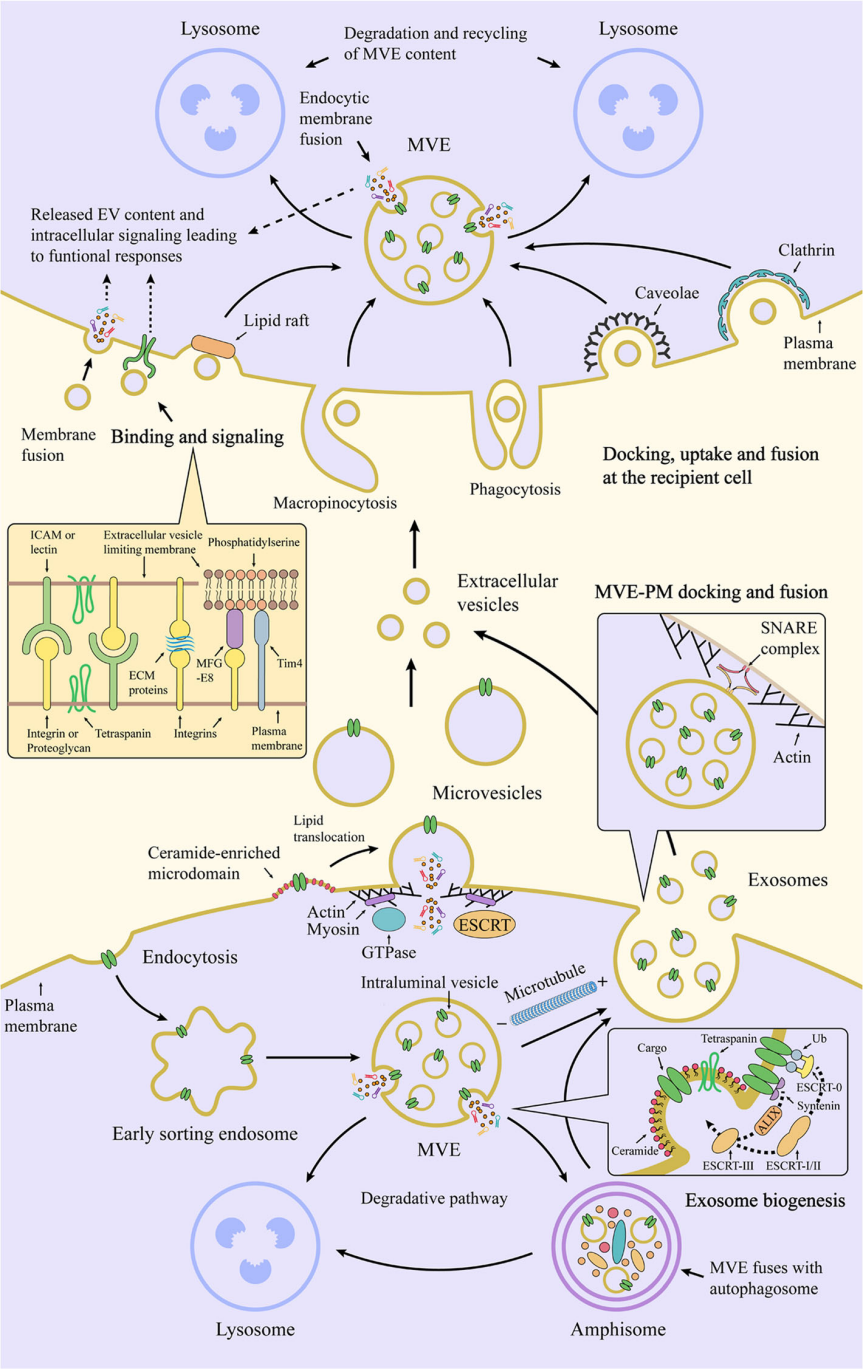
Extracellular vesicle biogenesis and secretion in donor cells as well as its interaction with and intracellular fate in recipient cells. Microvesicles directly shed from the plasma membrane, where budding microdomains undergo phosphatidylserine translocation and remodeling of the actin cytoskeleton. By contrast, exosomes originate from endosomal pathway. Deriving from endocytosis, early sorting endosomes accumulate ILVs within the endosomal lumen and then mature into MVEs, where ESCRT components, ceramide, tetraspanins and syntenin could act in parallel or separately to recruit exosomal cargoes and generate ILVs. At this checkpoint, the MVEs can either enter into autophagy-lysosome pathway or exosomal secretion pathway. of note, amphisomes can either fuse with lysosomes or the plasm membrane. Upon secretion into extracellular space, exosomes and microvesicles can bind to the recipient cell surface via ligand-receptor or glycoprotein interactions and initiate signaling, uptake and fusion processes, contributing to transfer functional messages and cellular phenotypes. MVE (multivesicular endosome), EV (extracellular vesicle), PM (the plasma membrane), Ub (ubiquitin), ECM (extracellular matrix), ESCRT (endosomal sorting complex required for transport), SNARE (soluble N-ethylmaleimide-sensitive factor attachment protein receptor)

Various types of cells are capable of secreting membrane vesicles, collectively termed extracellular vesicles (EVs), under both physiological and pathological states [1]. The amount and/or composition of released EVs change with external stimuli, such as pH, hypoxia and oxidative stress [2–4]. Based on their origin and size, EVs are broadly classified into two main classes: exosomes and microvesicles [5, 6]. Exosomes originate from intraluminal vesicles (ILVs) in multivesicular endosomes (MVEs), in which ILVs are generated by the inward budding and fission of endosomal membrane and then released upon fusion of MVEs with the plasma membrane (Fig. 1). Microvesicles, also called oncosomes in case of being released from cancer cells, shed directly from the plasma membrane or its extensions (for example, microvilli, filopodia) by an outward budding and fission (Fig. 1). Apoptotic bodies, derived from membrane blebbing during cell apoptosis, are another common subtype of EVs [7]. Although EVs were initially considered to dispose waste materials, their abilities in transferring cargoes between cells have attracted growing interests over the past decade [1]. The informative cargoes of EVs regulate biological functions at autocrine, paracrine and systemic levels and are transported in protected and directed manners to recipient cells. EV-mediated bidirectional communication between cells has played a key role in regulation of cancer initiation, development and progression [8, 9]. Increasing evidence indicates that enhanced EV secretion from cancer cells and dysregulation of their cargoes are associated with tumorigenesis [10]. Thus, tumor-derived EVs can serve as diagnostic and prognostic biomarkers of cancers as well as novel therapeutic targets and tools [11, 12]. Apart from proteins, metabolites and DNAs of EVs, EV-RNAs are also considered as important intercellular mediators affecting hallmarks of cancer [12]. Multiple RNA species are found in EVs, where non-coding RNAs (ncRNAs), particularly shorter RNA species, comprise the majority of EV-RNA transcripts [13]. The biological functions of these ncRNAs and their underlying mechanisms on recipient cells remain largely unknown and warrant further investigations. In this review, we summarize the cellular machineries and processes of EV formation, secretion and interaction with recipient cells; RNA sorting into EVs; biological roles of EV-ncRNAs, mainly including micro RNAs (miRNAs), long non-coding RNAs (lncRNAs) and circular RNAs (circRNAs), from various cells as well as their molecular mechanisms affecting phenotypes of recipient cells in premetastatic niches and the tumor microenvironment (TME).

### Extracellular vesicle biogenesis

EVs have different modes of biogenesis according to their origin—endosome and the plasma membrane. However, clustered membrane microdomains, certain sorting machineries, membrane invaginations and fission processes are essential for inward-budding vesicles at the limiting membrane of the secretory MVEs (exosomes) and an outward-budding vesicle at the plasma membrane (microvesicles) [5].

### Intraluminal vesicle generation and endosomal sorting in MVEs

Within the endosomal system, early sorting endosomes carry membrane cargoes that are internalized from the plasma membrane or originated from the *trans*-Golgi network, and then they mature into MVEs when ILVs accumulate within the lumen of endosomes [14]. The membrane cargoes could serve as regulators of selective recruitment of sorting machineries [15, 16]. Sorting machineries are required for cargo segregation on microdomains and subsequent inward budding and fission of ILVs.

The membrane remodeling role of the endosomal sorting complex required for transport (ESCRT) machinery in ILV biogenesis is of major importance to the formation of MVEs and exosome secretion. The siRNA-mediated depletion of multiple subunits of the ESCRT machinery and accessory proteins reveals their respective roles in modulation of the secretion and protein composition of exosomes [17]. Interestingly, simultaneous knockdown of key components of the ESCRT machinery still allows the formation of a few enlarged ILVs devoid of EGFR in distinct MVEs upon EGF stimulation [18]. As the best-characterized mechanism, the ESCRT machinery harbors four biochemically distinct protein complexes (ESCRT-0, −I, −II, and -III), which together with accessory proteins perform in a stepwise manner to sequester MVE cargoes in endosomes and induce the inward budding of endosomal membrane to form ILVs [19]. More specifically, early-acting ESCRT components segregate ubiquitinated membrane cargoes and probably initiate membrane bending on discrete endosomal microdomains, subsequently recruiting ESCRT-III; ESCRT-III subunits, together with the ATPase VPS4, further induce the budding and fission of the microdomains away from the cytosol [20].

In addition to ubiquitin-dependent endosomal sorting, syndecan–syntenin-ALIX axis modulates loading of ILVs with specific cargoes and production of the distinct subpopulations of exosomes. Cytosolic adaptor syntenin connects transmembrane protein syndecan to ESCRT accessory component ALIX, which could bridge the gap between syndecan and the ESCRT-III subunits, thereby facilitating the exosome secretion as well as exosomal release of syntenin, cleaved syndecans and syndecan cargoes (for example, FGF-FGFR complexes) [16]. Syntenin also interacts with CD63 on endosomal membranes and significantly influences exosomal release of CD63. Tetraspanin-6 is another syntenin-interacting membrane protein, and their interaction promotes exosome secretion [21]. The regulators of syndecan-syntenin-ALIX pathway include heparanase, small GTPase ADP ribosylation factor 6 (ARF6) and its effector phospholipase D2 (PLD2). Specifically, heparanase could induce efficient clustering of syndecans by trimming their heparan sulfate chains and allow enhanced binding of syntenin to endosomal syndecans, thereby promoting intraluminal budding and syntenin exosome secretion [22, 23].

The first exosome biogenesis pathway independent of ESCRT requires the generation of ceramide on MVEs [24]. With the cone-shaped structure and the self-association capability through hydrogen bonding, ceramide could induce a spontaneous curvature on the membranes and trigger the coalescence of ceramide microdomains into macrodomains or membrane platforms [25, 26]. Moreover, ceramide-dependent endosomal sorting requires activation of inhibitory G protein (Gi)-coupled sphingosine 1-phosphate (S1P) receptors on MVEs. Specifically, S1P, the ceramide metabolite, constitutively activates the Gi-coupled S1P receptors in an autocrine manner, thereby activating the Rho family GTPases Cdc42 and rac1 and forming F-actin networks on MVEs [27, 28]. Certain cargoes, such as proteolipid protein, CD63, CD81 and flotilin-2, are sorted into the ILVs of MVEs in a ceramide-dependent manner.

Proteins of the tetraspanin family have also been shown to mediate endosomal sorting, ILV formation and production of different exosome subpopulations. During melanogenesis, tetraspanin CD63 regulates a balance between the ESCRT-independent and -dependent endosomal sorting of the PMEL luminal domain, and Apolipoprotein E (ApoE) regulates the loading of the PMEL luminal domain into ILVs in the CD63-dependent sorting pathway [29, 30]. Interestingly, CD63 and Hrs mediate competing mechanisms that promote the formation of different sized ILVs [31]. Moreover, tetraspanin CD82 (and likely CD9) could form complexes with E-cadherin at the plasma membrane, which sorts cellular β-catenin to MVEs and exosomes [32].

Collectively, cargo sorting and ILV budding in MVEs are closely-related processes and mediated by both ESCRT-dependent and ESCRT-independent pathway. It is plausible that in these processes, different pathways can function on same or different MVEs, and collaborative pathways can participate in different steps or certain steps. Hence, different MVE or ILV subpopulations could coexist and contain distinct compositions and morphology.

### Intracellular fate, transport and extracellular release of MVEs

The matured MVEs are targeted either to lysosomes or autophagosomes for the degradation and recycling of their contents or to plasma membrane for release of ILVs, referred as exosomes (Fig. 1**)**. Although the main fate of MVEs is to fuse with lysosomes, the balance between the degradative and secretory pathways of MVEs could be reversed, resulting in increased exosome secretion. The exosome secretion is considered as a homeostatic response to counteract fluctuant lysosomal or autophagic activity [33–35]. Due to the impaired autophagy and lysosomal pathways, MVEs with superfluous or defective cargoes, such as cholesterol and self-aggregating proteins could be rerouted to the plasma membrane for exocytosis [36, 37] Notably, there is another scenario that defected autophagic and lysosomal functions promote loading of degradative cargoes into ILVs and exosomes but do not increase exosome secretion, suggesting the MVE fate remains unchanged [38]. Macroautophagy starts with sequestration of waste or damaged cellular components into autophagosomes, and they can fuse with MVEs to form amphisomes, which can subsequently fuse with lysosomes [39]. Upon inhibition of degradation, amphisomes can reroute to the plasma membrane and extracellularly release their ILVs with autophagy-associated proteins (Fig. 1). Altogether, autophagy-exosome and lysosome-exosome crosstalks can influence the fate of MVEs and their cargoes towards secretion or degradation. The underlying mechanisms of this balance are still under investigation but possibly involve the components of the exosome biogenesis and autophagy pathway. ISGylation of TSG101, one of ESCRT-I components, promotes TSG101 aggregation and degradation by inducing MVE fusion with lysosomes, thereby repressing MVE numbers and exosome secretion [40]. Tetraspanin-6 increases exosome secretion by activating syntenin pathway, which is correlated with impaired autophagosome-lysosomal fusion [21]. Prion protein induces caveolin-1 (CAV-1) internalization and subsequent inhibition on ATG12–ATG5 complex, leading to autophagy inhibition and exosome secretion [35]. Alcohol-induced miR-155 disrupts autophagic and lysosomal functions to enhance exosome secretion by targeting LAMP1 and LAMP2 [34]. SIRT1 promotes ATP6V1A mRNA stability and then disrupts function of V-ATPase proton pump, thereby reducing lysosomal acidification and increasing exosome secretion [41]. PIKfyve depletion, which inhibits PI(3,5)P2 synthesis, reduces autophagic flux and autophagic degradation, which consequently promotes the secretion of exosomes containing autophagy-related proteins [42]. In addition, independent of controlling autophagy–exosome balance, autophagy machineries have recently been reported to modulate MVE formation and targeting. ATG12-ATG3 axis promotes exosome secretion, endolysosomal trafficking and basal autophagy by interacting with Alix and thereby inducing its active ‘open’ conformation [43]. Independent of ATG7 and canonical autophagy, ATG5 decreases acidification of MVEs by removing ATP6V1E1 from V1V0-ATPase, thereby increasing exosome production [44].

As for MVEs destined for secretion or degradation, their intracellular transport, docking and final fusion with intracellular membranes are indispensable and tightly regulated. MVEs are transported along cytoskeleton to the membranes, which is modulated by multiple molecular motors and switches (small GTPases )[45, 46]. Various Rab GTPases, which shift from GDP- to GTP-bound states for activating effectors, participate in modulation of MVE targeting. Rab7-dependent transport of MVEs to lysosomes has been well documented, and exosome secretion could depend on ubiquitylation status of Rab7 and endosomal cholesterol levels, which modulate dynein motor-mediated MVE transport [47–49]. Moreover, Rab24 is involved in Rab7-mediated endolysosomal degradation possibly by interacting with Rab7 and its effector RILP [50]. In the transport and docking of MVEs, Rab27a and Rab27b perform different roles at distinct locations possibly by activating their respective effectors Slp4 and Slac2b [15]. Moreover, Rab11 and Rab35-induced releases of exosomes rely on intracellular Ca^2+^ levels. Munc13–4, as a Rab11a effector, promotes trafficking of Rab11^+^ endosomes to CD63^+^ MVEs, in order to increase the size and secretion competence of MVEs [51]. Rab35, mediated by TBC1D10A–C, acts on the plasma membrane for docking or tethering of MVEs [52].

Depending on the cell types and cellular status, MVEs are transported towards the plasma membrane for multidirectional secretion or polarized secretion [53]. The delivery of MVEs to specific membrane locations depends on microtubule and branched actin network, as reported for immune synapse (IS) between immune cells and invadopodia in cancer cells [54, 55]. The oriented transport of MVEs is controlled by positioning of the microtubule-organizing center (MTOC), which is redirected towards IS during antigen presentation. The MTOC reorientation requires phospholipase C-γ-mediated accumulation of diacylglycerol (DAG) at the IS [56]. In lymphocytes, DAG kinase α, transforming DAG into phosphatidic acid, serves as a negative regulator of maturation and polarized traffic of MVEs by reducing PKD1/2, DAG effector, recruitment to and activation at the IS [57, 58]. Within invadopodia, cortactin binds with the Arp2/3 complex to promote actin nucleation, which stabilizes the branched actin and allows more docking sites of MVEs at the plasma membrane [59].

As the final step of exosome release, MVE fusion with the plasma membrane is governed by soluble N-ethylmaleimide-sensitive factor attachment protein receptor (SNARE) proteins and their regulators. Membranes have their distinct set of SNARE proteins, target-membrane SNAREs (t-SNAREs) and vesicle-membrane SNAREs (v-SNAREs) anchored to the acceptor and vesicle membranes respectively, and their pairing and assembly into the SNARE complexes bring the membranes into close proximity, thereby driving membrane-fusion events. As a v-SNARE, VAMP7 is required for exosome secretion, owing to its ability to modulate MVE fusion with the plasma membrane [60]. The exosome secretion of tumor cells has been shown to rely on PKM2-mediated and H1HR-mediated phosphorylation of SNAP23, which could promote the formation of the SNARE complex to facilitate the docking and fusion between MVEs and the plasma membrane [61, 62]. Other SNARE proteins also participate in exosome secretion, such as Ykt6 and VAMP5 [63, 64]. Notably, GTPase RAL-1 regulates not only the formation of MVEs, but also their fusion with the plasma membrane by colocalizing with t-SNARE SYX- 5[65].

### Microvesicle biogenesis and release

Compared with exosome biogenesis, microvesicle biogenesis is not fairly well understood. Microvesicle release can be initiated by increased Ca^2+^ concentration, which results in disruption of membrane asymmetry and actin cytoskeleton rearrangements by mediating Ca^2+^-dependent enzymes [66]. This enzymatic pathway includes flippases, floppases and scramblases that mediate the translocation of phosphatidylserine from the inner leaflet to the cell surface, as well as calpain and gelsolin that cleave actin filaments and capping proteins respectively [67]. Moreover, Peptidylarginine deiminases (PADs), Ca^2+^-dependent enzymes, convert protein-bound arginine to citrulline for deiminating proteins; PADs stimulate microvesiculation through deimination of cytoskeletal actin [68]. Loss of membrane lipid asymmetry imposes local membrane curvature during microvesicle formation, followed by actin-myosin-based contraction that promotes microvesicle fission and release [69]. Of note, phosphatidylserine exposure does not occur in entire microvesicle population, suggesting the involving of other mechanisms in microvesicle budding, including clustering of transmembrane proteins with spontaneous curvature as well as changes in lipid composition (for example, cholesterol and galactosylsphingosine) and related domains [70]. The releasing process of microvesicles requires ATP-dependent actomyosin contractile machinery composed of actin and myosin, which facilities contraction at microvesicle necks [71]. Interestingly, this contractile machinery propels sliding of apical membrane towards the microvillus tip and leads to membrane vesiculation and microvesicle shedding at the tip [72, 73].

The transmembrane protein TMEM16F, which has scramblase activity, induces phosphatidylserine exposure and platelet-derived microvesicle release [74]. The ARF and RHO families of small GTPases are important regulators of actin dynamics and facilitate actin cytoskeleton-based fission of microvesicles in tumor cells. ARF1 modulates the activation of RhoA and RhoC, which leads to myosin light-chain (MLC) phosphorylation and actomyosin contraction [75]. Activation of RhoA, and its downstream effector RHO-associated protein kinase (ROCK), has been shown to activate Lim kinase (LIMK) that can phosphorylate cofilin and inhibit its actin-severing activity [76]. In addition to participating ARF1/Rho/MLC and RhoA/ROCK/LIMK/cofilin signaling, RhoA/ROCK signaling activates extracellular signal-regulated kinase (ERK) and then inhibits myosin light chain phosphatase (MLCP), which inactivates MLC, thereby promoting microvesicle secretion; and this Rho signaling are promoted by ARF6 activation and Rac1 downregulation [77]. Interestingly, activation of ARF6 promotes phosphorylation of MLC by activating phospholipase D (PLD) and then recruiting ERK to the plasma membrane for phosphorylating MLC kinase (MLCK), resulting in microvesicle release; whereas its inactivation induces the opposite effect through PKC-mediated phosphorylation of MLC and then decreased MLC activity [71]. These observations suggest that ARF6 harbors two distinct downstream pathways (RhoA/ERK/MLCP/MLC and PLD/ERK/MLCK/MLC) to promote MLC activity and microvesicle shedding. Apart from the small GTPases, other cytoskeletal regulators are available for microvesicle secretion. DIaPh3 suppresses membrane bleb formation and microvesicle secretion, which is associated with phosphorylation state of cofilin [78]. Activation of protease activated receptor 2 (PAR2) by trypsin induces AKT phosphorylation and then activates Rab5a at the plasma membrane, resulting in polymerization of actin and microvesicle secretion [79]. In a later report, Activation of PAR2 regulates actomyosin rearrangements to enhances microvesicle secretion via three independent pathways, including MAPK/MLCK/MLC, P38/MK2/HSP27 and RhoA/ROCK signaling [80].

Additional cell surface receptors are involved in microvesicle secretion, including G protein-coupled receptor 30 [81], α-2-Macroglobulin receptor [82], transient receptor potential vanilloid type 1 [88] and tissue factor [83]. In addition, microvesicle secretioin is also induced by intratumoral hypoxia, which transcriptionally regulates the expression of the small GTPase Rab22A that colocalizes with budding MVs [84]. Glycosaminoglycan on the cell surface also participates in microvesicle secretion. Hyaluronan synthase 3 (HAS3), which synthesizes hyaluronan on the plasma membrane, induces microvesiculation at the tips of microvilli and secretion of HAS3- and hyaluronan-positive microvesicles [85].

The biogenesis and release of microvesicles have shared partly common machineries with exosomes, such as ESCRT proteins and ceramide. Membrane-associated arrestin­domain­containing protein 1 (ARRDC1) recruits TSG101 to the plasma membrane and then drives the release of ARRDC1­mediated microvesicles (ARMMs), and the ATPase VPS4 is also involved in such release [86]. Upon ATP stimulation, acidic sphingomyelinases translocate to the outer leaflet of the plasma membrane and then generate ceramide to promote membrane evagination and microvesicle shedding [87]. Mechanistically, this process depends on ATP-mediated activation of P2X7 receptors that induces p38 MAPK cascade through src kinase.

### EV biogenesis and release in cancers

Regulators of EV secretion have been shown to be overexpressed or activated in various cancers, including ESCRT components, syntenin, heparanase, small GTPases (such as Rab27A and Rab27B), SNARE proteins (such as SNAP23) [10, 62, 89]. The elevated activities of the EV regulators could explain the increased secretion of EVs from cancer cells compared with their normal counterparts.

Compared with normal cells, cancer cells could adapt distinct pathways to enhance EV secretion in terms of the influence of oncogenes (such as EGFRvIII and H-RAS^V12^). The signaling pathways of oncogenic secretion of EVs have been revealed. For example, the proto-oncogene SRC promotes the release of promigratory exosome by phosphorylating syndecans and syntenin [90]. As another example, v-H-Ras enhances microvesicle secretion by inducing ERK-dependent CSE1L phosphorylation [91]. The ncRNAs also involve in the EV secretion of cancers. For example, lncRNA HOTAIR promotes transport of MVEs to the plasma membrane and exosome secretion by mediating Rab35 and SNAP23 [92]. lncRNA HULC increases exosome secretion by regulating miR-372-3p/Rab11a axis [93]. miR-200a could stabilize the polymerized actin networks and suppress microvesicle secretion by targeting gelsolin [94].

### Binding, fusion, internalization and fate of extracellular vesicles upon their interactions with recipient cells

As mediators of intercellular communication, EVs can travel through the extracellular space and dock to recipient cells, resulting in delivery of their contents or signals (Fig. 1). Upon docked at recipient cell membranes, EVs can activate surface receptors of the recipient cells or release their cargoes via internalization or fusion with the recipient cells [95]. The modes of vesicle internalization include phagocytosis, macropinocytosis as well as lipid raft-dependent, clathrin-dependent and caveolae-dependent endocytosis [96]. Upon endocytic uptake, endocytosed vesicles can enter endosomal system and probably coexist with endogenous ILVs in MVEs; the internalized vesicles can be targeted to lysosomes or fuse directly with endocytic membrane to release their intraluminal material, thereby leading to recycling of their cargoes and transferring functional molecules into cytoplasm, respectively [5]. Fusion of EVs with the plasma membrane also allows the release of intraluminal contents with functional responses [97].

The targeting of EVs to recipient cells, which can be the producing cells themselves, or organs is specific both in vitro and in vivo and depends mainly on specific interactions between proteins on the surface [98]. Multiple mediators participate in these interactions and may be required for downstream signaling and processes, including tetraspanins, extracellular matrix (ECM) proteins, integrins, proteoglycans, lectins and lipids. Exosomal tetraspanins could selectively recruit other membrane proteins, such as integrins, and then form tetraspanin-complexes, thereby promoting exosome docking and uptake by target cells [99, 100]. ECM proteins on EVs, such as fibronectin and laminin, interact with integrin on the cell surface to promote cellular docking and uptake of the EVs and to activate integrin-mediated signaling events in recipient cells [101, 102]. In addition, integrins on EVs can also bind to adhesion molecules, such as ICAMs, expressed on recipient cells, and vice versa [103, 104]. Intriguingly, integrin-associated CD47 on EVs prevents phagocytic clearance of the EVs from circulating monocytes by binding SIRPα [105]. The specific interactions also rely on heparan sulfate proteoglycans and lectins, and they have been shown to present at the surface of EVs and target cells and to bind to each other [106–108]. Altogether, adhesion molecules have been shown to have key roles in the membrane interaction between EVs and acceptor cells. Other molecules such as phosphatidylserine, exposed at the surface of EVs, interact with lipid-binding proteins such as MFGE8 and TIM4 that allow capture of the EVs by selected recipient cells [109, 110]. Notably, tetherin attaches and clusters EVs on the plasm membrane of producing cells [111].

### Machineries involved in the RNA sorting into extracellular vesicles

**Fig. 2:**
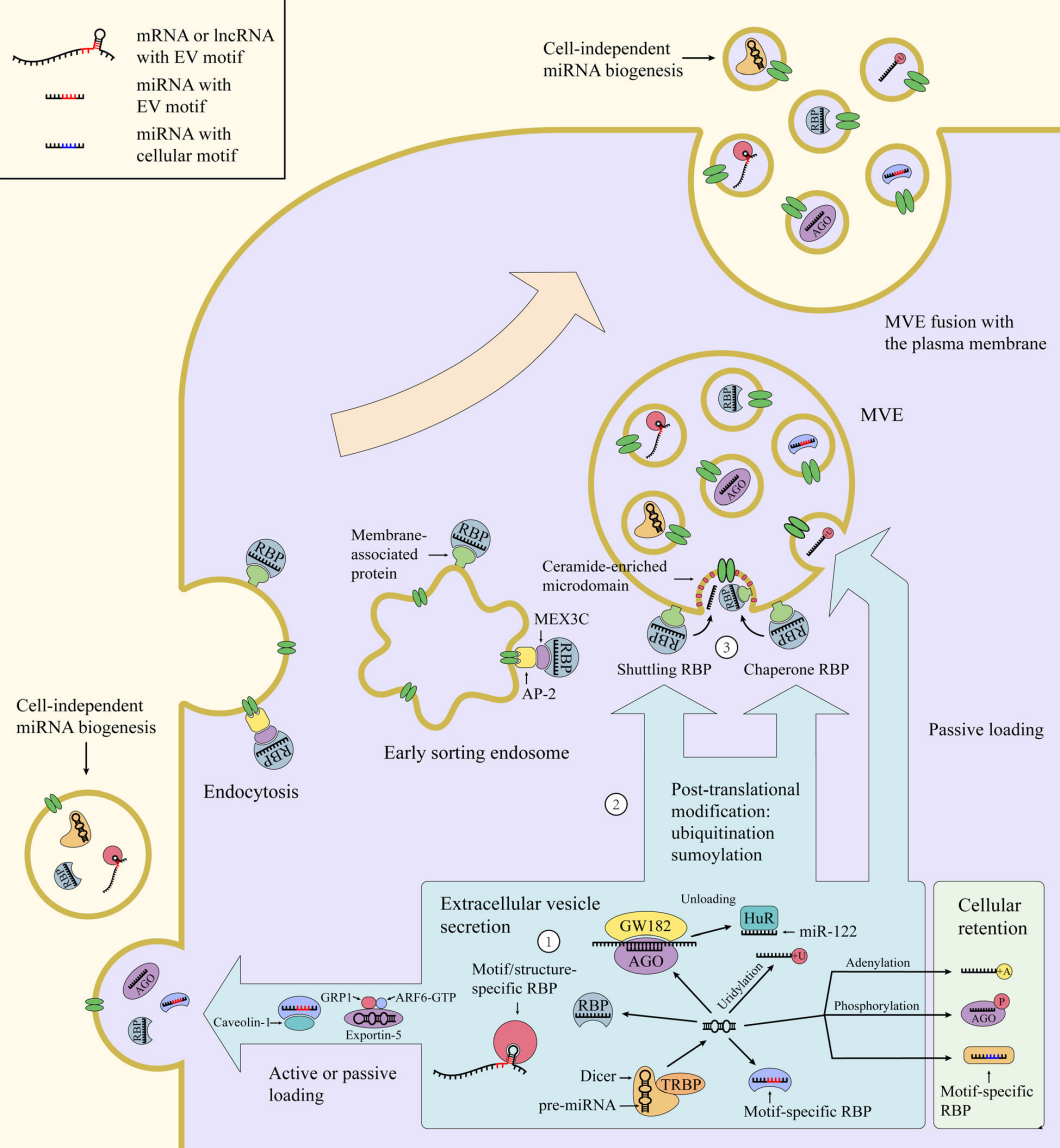
RNA incorporation into EVs. Various RBPs and membrane-associated proteins are required for the different steps of EV-RNA sorting. First, intracellular RNAs can interact with RBPs or motif-specific RBPs, which may prevent RNAs from degradation. Second, RNA-loaded RBPs undergoing post-translational modification can be recruited to the sites of EV budding via binding to membrane-associated proteins; otherwise, RNAs and RNA-loaded RBPs are incorporated passively into EVs. Third, upon reaching the budding membrane, the RBPs can be co-sorted with loaded RNAs into EVs or unload RNAs into EVs. In addition, 3′ end RNA tailing, such as adenylation and uridylation, controls RNA distribution between cells and EVs. MVE (multivesicular endosome), EV (extracellular vesicle), RBP (RNA binding protein).

Intracellular RNAs are kept in close proximity to site of EV biogenesis and then incorporated into the EV lumen, which is affected by affinity of RNAs and their carriers to membrane lipids and proteins at the budding microdomain [112] (Fig. 2). Discordant enrichment of RNAs in EVs indicates that incorporation of RNAs into EVs is actively regulated by particular sorting machineries, although the RNA loading can occur in a random manner [113, 114]. These machineries are likely to involve RNA binding proteins (RBPs) and their associated partners, which can target RNAs to the site of EV generation and protect them from degradation. Current studies have mostly focused on regulators of miRNA sorting into exosomes, including those related to miRNA biogenesis and function as well as the exosome biogenesis. Dicer depletion has a stronger inhibitory effect on miRNA levels in the exosomes than in the producer cells, whereas miRNA overexpression increases miRNA levels to a greater extent in the exosomes than in the cells [115], suggesting that the expression level of miRNAs is the first layer of regulation of miRNA sorting into exosomes. The main components of miRISCs (miRNA-loaded RNA-induced silencing complexs), such as Agos and GW182, often colocalize with MVEs, turnover of which regulates miRNA loading onto miRISCs and miRISC activity [116, 117]. Through association with endosomal pathway, miRISCs could mediate RNA-silencing processes and influence the intracellular locations of pools of miRNAs and miRNA-repressible transcripts. In this context, it was reported that levels of target transcripts control miRNA sorting to exosomes via their interactions [115]. Furthermore, Ago2 knockout reduces loading of several preferentially secreted miRNAs into EVs, such as miR-451 and miR-15 0[118]. Upon loss of Ago2, highly secreted miR-451 is the most affected, probably because its Dicer-independent maturation requires only Ago2-mediated cleavage. Ago2 knockdown also decreases the exosomal content of small RNAs, indicating that Ago2 may serve as an important transferring machinery for EV-miRNAs [119]. This notion was further strengthened by the fact that Ago2 can be sorted to exosomes and control the sorting of specific miRNAs (for example, let-7a) into exosomes. However, the presence of Agos in EVs is still questionable as the regulation of Agos on endosomal membranes may differ according to cell type or cellular state [112]. GW182 knockdown decreases the release of exosomal miRNA, such as miR-146a and miR-155, probably by making Ago-loaded miRNAs more vulnerable to ribonucleas e[120]. Interestingly, as a negative regulator of miRNA function, HuR can replace Ago2 from target mRNAs and capture miR-122 from Ago2, and ubiquitination of HuR on MVEs promotes miR-122 unloading and then the extracellular export of miR-122 [121].

In addition, ESCRT proteins have been shown to regulate miRNA sorting into EVs. Alix knockdown reduces loading of secreted miRNAs into EVs but not the release of EVs probably by interacting with Ago2 [122]. However, the other study reported that Alix knockdown does not influence the extracellular export of miR-146a [123]. Another ESCRT protein, Vps4A, mediates the release of oncogenic miRNAs in exosomes [124].

Additional RBPs have been shown to be involved in the sorting of specific miRNAs into EVs. Upon nuclear export and dissociated from Ran-GTP, Exportin-5 dictates pre-miRNA complex by interacting ARF6-GTP-GRP1 complex that transfers miRNAs to microvesicle biogenesis sites [125]. MVP presents in exosomes and promotes the sorting of miR-193a into exosomes via binding to miR-193a [126]. YBX1 interacts with miR-223 and promotes miR-223 sorting into exosomes, and it is also involved in TICAM-1-mediated sorting of miR-21 into EVs [127, 128]. MEX3C can be targeted to endolysosomal pathway through interaction with AP-2 complex and associated with an RBP of miR-451a, thereby allowing the sorting of miR-451a into exosomes [129].

Certain RBPs have been proposed to perform miRNAs sorting by recognizing specific RNA motifs. hnRNPA2B1 controls exosomal sorting of miRNAs with the GGAG motif, and sumoylation of hnRNPA2B1 promotes its binding to the miRNAs and localization into exosomes [130]. Cav-1, a membrane-bound protein, forms complex with hnRNPA2B1 and induces hnRNPA2B1 O-GlcNAcylation via its tyrosine-14 phosphorylation, thereby directing hnRNPA2B1-bound miR-17/93 into microvesicles; O-GlcNAcylation of hnRNPA2B1 enhances its binding to specific miRNAs and incorporation into microvesicles [131]. Similarly, SYNCRIP displays the GGCU-motif-specific exosomal sorting capacity for miRNAs [132]. By contrast, ANXA2 mediates the sorting of miRNAs into EVs in a sequence-independent manner and binds EV miRNAs in the presence of Ca^2+^ [133].

In addition to RBPs, sphingomyelinase pathway has been shown to involve in exosomal export of miRNAs. Inhibition of neutral sphingomyelinase 2, and therefore the ceramide generation, prevents the sorting of multiple miRNAs into EVs, such as miR-451a, miR-122 and miR-146a [121, 123, 129]. As another example, inhibition of sphingosine kinase 2, and therefore Sphyngosine-1-phosphate generation, reduces exosomal loading of miRNA-21 [134].

Selection of miRNAs for exosomal release is also tightly associated with their 3′ end post-transcriptional modifications. miRNAs distribution relies on 3′ end uridylation and adenylation, which promotes miRNAs exosomal release and cellular retention respectively [113]. For example, in cancer cells, miR-2909 is targeted to or excluded from exosomes in a manner dependent on its 3′-end adenylation to uridylation ratio, which seems to be linked to different distribution of adenosine kinase between cells and exosomes [135].

Although it is still lack of evidence that how lncRNAs are targeted to EV production site, they are likely to share common *cis*-acting signals and sorting machineries (*trans*-acting proteins) with mRNAs. mRNAs have been shown to differentially sorted to EVs mostly depending on their specific sequences and secondary structures in the 3′-untranslated regions. The presence of three motifs (ACCAGCCU, CAGUGAGC and UAAUCCCA) in mRNAs and lncRNAs is associated with their exosomal secretion, and YBX1 could be involved in the sorting process by specifically binding with these motifs [136–138]. Interestingly, miRNAs could also regulate mRNA targeting into EVs by specifically binding with zipcode RNA sequence motifs. miR-1289 directly binds with the inserted zipcode on EGFP mRNA and then enhances the efficiency of zipcode-mediated EGFP mRNA sorting into microvesicles [139]. The potential roles of miRNAs in transferring mRNAs into EVs are also illustrated by the fact that miRNA binding sites are found in the predicted motifs enriched in EV mRNAs [140].

RNA content of EVs varies depending on the EV subpopulation, cell type and the physiological or pathological state of producing cells as well as their received stimuli. Origin of EV-RNA diversity can be attributed to cellular RNA profile and different RNA sorting and protection mechanisms. EV-RNA loading can occur by either active or passive mechanisms and largely depend on RBPs and their partners as well as RNA motifs and modifications, with combined effect on stabilization and/or subcellular localization of EV-RNAs. Chaperone RBPs can be co-sorted with intracellular RNAs and present on exosomes, whereas shuttling RBPs can transfer RNAs to membrane-bound RBPs in MVEs and exclude themselves from exosomes. Post-translational modification of RBPs is associated with their affinity for MVEs and RNAs, suggesting an additional layer of regulation of exosomal sorting. RBP-mediated RNA incorporation into EVs has been shown to depend on ceramide generation, indicating RBPs are likely to be recruited to the ceramide-enriched microdomains that will bud in selective RNA-loading processes. 3′-end of RNA sequence appears to be a primary site that contains RNA sorting signals for EV secretion. Specific motifs and structures of RNAs play important roles in EV-RNA secretion by mediating RNA-RBP and RNA-RNA interaction. Nontemplated nucleotide additions have an impact on RNA distribution between EVs and cells probably by controlling RNA metabolism.

### Biological roles of RNA-containing extracellular vesicles in the TME and premetastatic niches

**Fig. 3:**
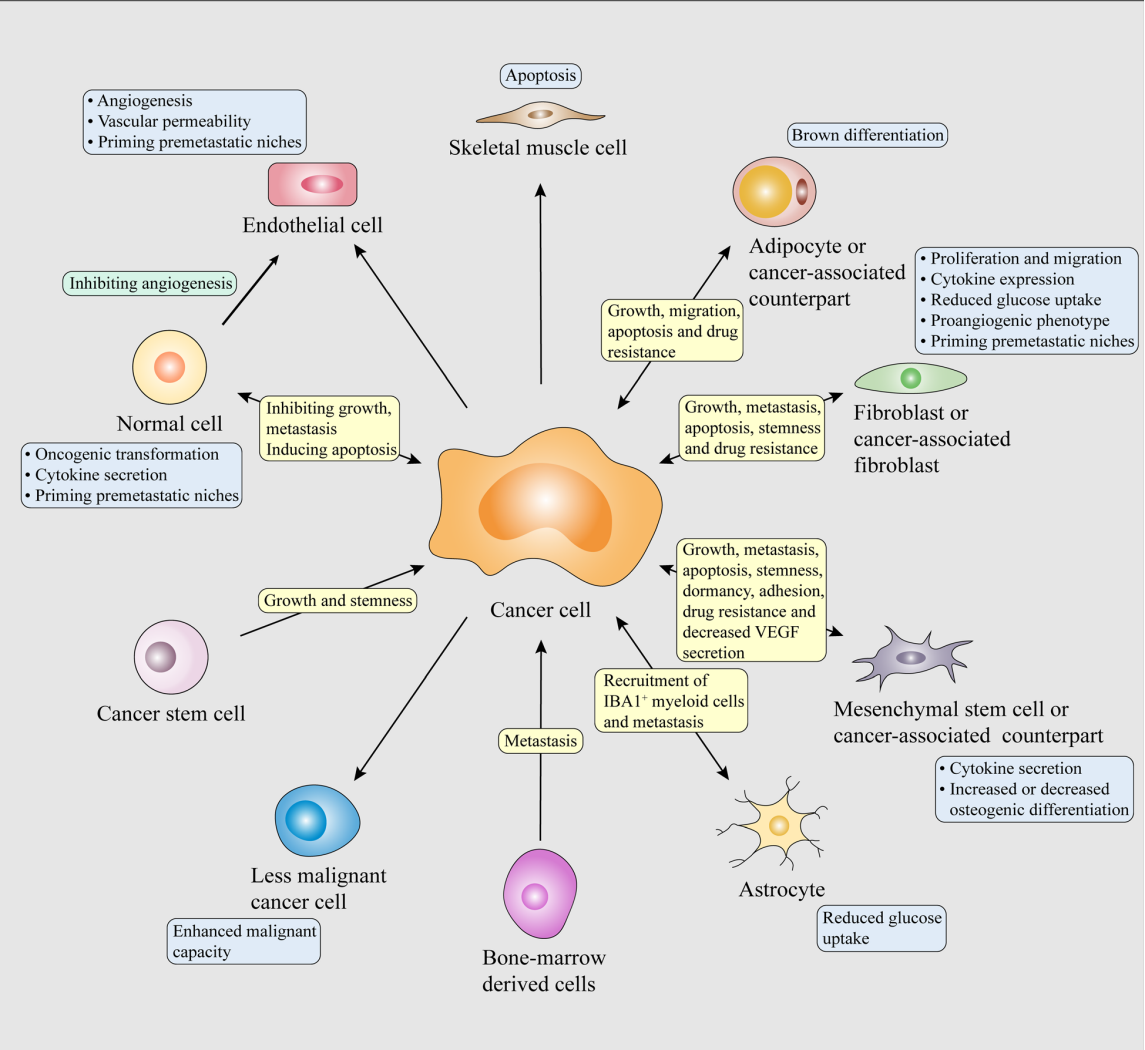
EV-RNA mediated crosstalk within tumors and between tumors and stroma modulating malignant behaviors of cancer cells. Cancer initiation, development and progression are attributed to sophisticated and multidirectional communication between various cells. Tumor-derived EV-RNAs can elicit oncogenic, prometastatic, proangiogenic and differentiated phenotypes of stromal cells in the tumor microenvironment or prometastatic niches. Tumor-derived EV-RNAs also drive normal and tumor cell subpopulations towards malignant phenotypes. EV-RNAs from cancer-reprogrammed stromal or normal cells also contribute to malignant behaviors of cancer cells, thereby affecting the growth, migration, invasion and survival of primary and metastatic cancer cells. Of note, EV-RNAs from normal cell can also restrain the malignant behaviors of cancer cells.

**Fig. 4:**
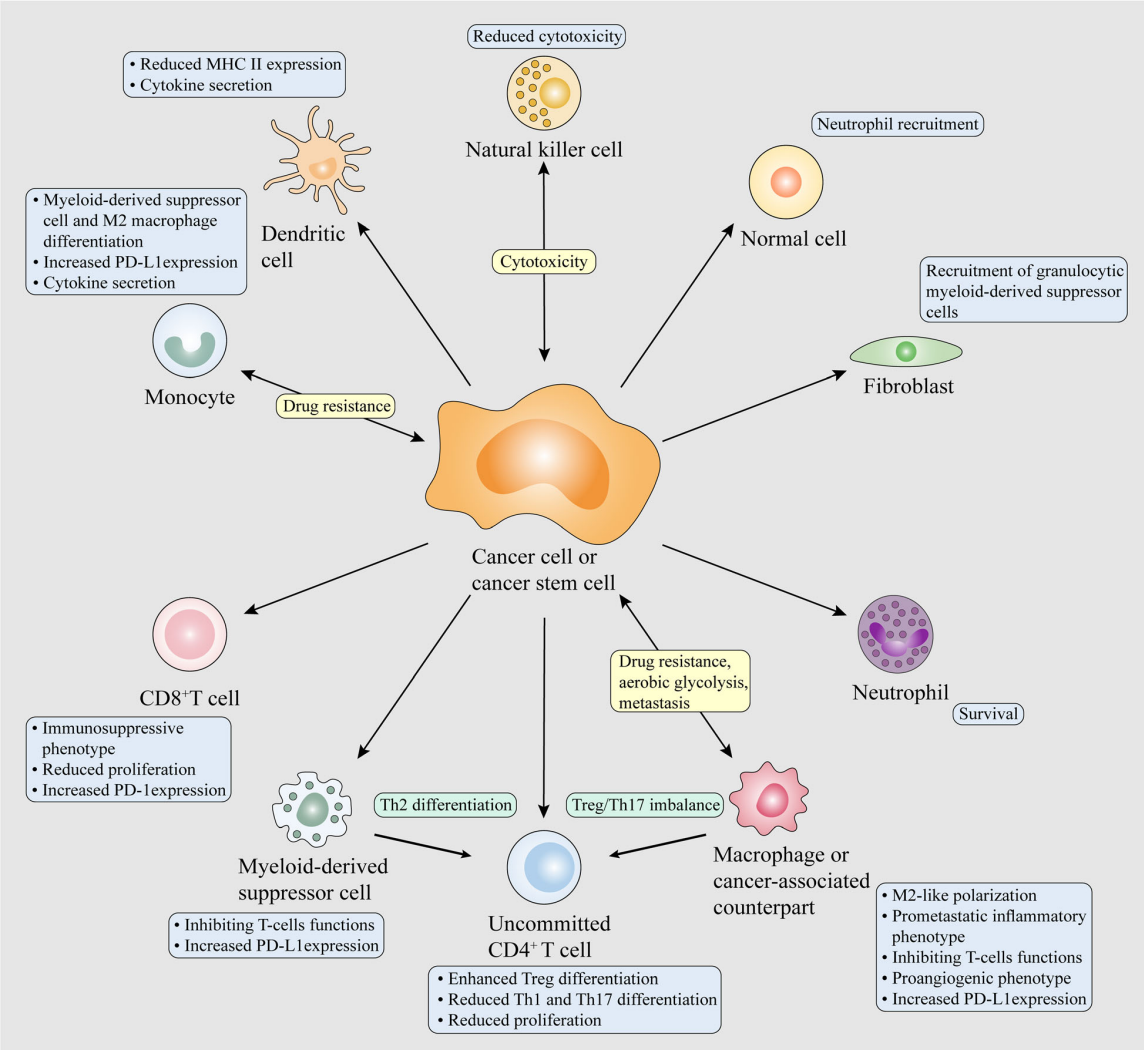
EV-RNA mediated crosstalk between cancer cells and immune cells and within immune cells modulating malignant behaviors of cancer cells. Tumor-derived EV-RNAs can contribute to the immunosuppressive and decreased anti-tumoral activities of various immune cells and induce immunoinhibitory phenotype of CAFs and normal cells. EV-RNA-mediated communication between immunes also leads to cancer progression.

The deregulation of EV-RNAs among different cancer types and their cell-type-specific functions have recently started to be uncovered. These EV-RNAs carry genetic messages of donor cells to neighboring or distant stromal and tumor cells, and contribute, at least in part, to bidirectional communication within the TME. Once reaching the recipient cells, EV-RNAs can trigger molecular and phenotypic reprogramming of recipient cells. The underlying mechanisms of EV-RNAs affecting cellular functions are different according to the type of RNA. In recipient cells, mRNAs delivered by EVs can be translated into functional proteins, whereas ncRNAs delivered by EVs can engage complex networks of ncRNA interactions and serve as important regulators of gene expression in cellular processes [141, 142]. EV-RNAs have been considered as oncogenic drivers or tumor suppressors in various types of cancers. The proliferation, apoptosis, migration, invasion, dormancy, stemness and therapy resistance of cancer cells are actively mediated by EV-RNAs from their malignant counterparts, educated noncancerous cells and normal cells (Figs. 3 and 4). EV-RNAs from cancer cells also continuously reprogram stromal cells to support tumor development and progression, establishing a feed-forward or -back loop of intercellular communication (Figs. 3 and 4). The reprogramming of stromal cells and immune cells results in stromal activation, vascular restructure and immune evasion, further driving tumor growth, invasion, metastasis and therapy resistance.

### Regulation of malignant phenotypes of cancer cells by tumor and stromal EV-RNAs

During cancer development, there are cell competition between cancer cells and neighboring normal cells [9]. As a homeostatic mechanism, abundant noncancerous cells can release tumor-suppressive miRNAs to inhibit malignant phenotypes of adjacent cancer cells [12]. EV-miR-143 from normal epithelial prostate cells suppresses the proliferation of adjacent prostate cancer cells [143]. EV-miR-145 from tumor-associated stroma cells impairs the viability and induces the apoptosis of adjacent pancreatic ductal adenocarcinoma cells [144]. EV-miRNAs from liver stem cells inhibit the proliferation and promote the apoptosis of hepatocellular carcinoma (HCC) cells in vitro and in vivo [145]. EV-miR-145 from adipose tissue-derived mesenchymal stem cells (AMSCs) inhibits the proliferation and promotes the apoptosis of prostate cancer cells [146]. EV-lncRNA-PTENP1 from normal cells induces the apoptosis of bladder cancer cells and inhibits their proliferation, migration and invasion by targeting miR-17, thereby reducing tumor growth in vivo [147]. Of note, EV-RNAs from normal cells also contribute to the malignant behaviors of cancer cells. EV-circRNA-DB from adipocytes promotes the proliferation and migration of HCC cells and reduces their DNA damage by targeting miR-34a, resulting in tumor growth and metastasis in vivo [148]. Mesenchymal stem cells (MSCs) are multi-potent stromal cells derived from bone marrow, adipose tissue, umbilical cord or placental, with potential to act as protumoral components of the TME under both normoxic and hypoxic states. EV-miR-410 from umbilical cord MSCs promotes the proliferation and inhibits the apoptosis of lung adenocarcinoma cells by targeting PTEN in vitro and in vivo [149]. EV-miRNAs, including miR-193a-3p, miR-210-3p and miR-5100, from hypoxic bone marrow mesenchymal stem cells (BMSCs) promote the mesenchymal-to-epithelial transition (EMT), migration and invasion of lung cancer cells by activating STAT3 pathway [150]. EV-miR-21-5p from hypoxic BMSCs increases the proliferation, survival, migration and invasion of lung cancer cells as well as M2 macrophage polarization, a protumorigenic shift, by targeting PTEN, PDCD4 and RECK, leading to tumor growth and intratumoral angiogenesis in vivo [151]. EV-miR-142-3p from BMSCs promotes the stemness, doxorubicin resistance, invasion and adhesion of colon cancer cells by targeting Numb [152]. EV-miR-23b from BMSCs reduces the proliferation, sensitivity to docetaxel and CD44, a stem cell marker, of bone marrow–metastatic breast cancer (BC) cells by targeting MARCKS, thereby contributing to dormancy of BC stem cells in metastatic niches [153].

The EV-mediated transfer of tumor-suppressive miRNAs to cancer cells indicates that manipulation of EV-RNAs could have therapeutic benefits in cancers. For example, stellate cell-derived EVs loaded with miR-335-5p inhibit the proliferation and invasion of HCC cells and HCC tumor growth in vivo [154]. In another case, EVs from miR-195-transfected fibroblasts induce tumor shrinkage and improve the survival in a rat cholangiocarcinoma model [155]. In addition, EVs from miR-122-transfected AMSCs sensitize HCC cells to sorafenib both in vitro and in vivo [156]. EVs from miR-126-3p-transfected BMSCs suppress the proliferation, migration, and invasion and increase the apoptosis of pancreatic cancer cells by targeting ADAM9, reducing tumor growth in vivo [256]. EVs from miR-138 -transfected γδ T cell inhibit the proliferation and promote the apoptosis of oral squamous cell carcinoma cells by downregulating GNAI2, FOSL1, CCND1, and CCND3, thereby reducing growth of tumor in xenograft-bearing nude mice; the miR-138-overexpressing EVs also promote the proliferation, interferon-γ secretion and cytotoxicity of CD8^+^ T cells by downregulating PD-1 and CTLA-4, thereby impairing tumor growth in immunocompetent C3H mice [157]. This study indicates that miR-138-overrxpressing EVs from γδ T cells harbor both anti-tumor and immune-stimulated effects.

Once cancer cells overcome the homeostatic response, the local and/or distant microenvironment are actively remodeled to support cancer development and progression. The intercellular transfer of tumor-promoting RNAs reprograms normal cells to co-evolve with cancer cells, thereby enabling a pro-cancerous crosstalk between cancer cells and noncancerous cells. Cancer-associated fibroblasts (CAFs), a predominant cell type in the TME, are activated from quiescent fibroblasts undergoing myofibroblast differentiation and may derive from MSCs. EV-miR-105 from cancer cells activates a MYC-dependent metabolic reprogramming by targeting MXI1 in CAFs, thereby assisting in tumor growth in different nutrient conditions [158]. EV-miR-211 from melanoma cells reprograms primary fibroblasts into CAFs by targeting IGF2R, thereby potentially fostering dermal tumor niches and melanoma invasion [159]. EV-miR-9 from BC cells promotes the acquisition of a CAF-like phenotype in normal fibroblasts (NFs). EV-miR-9 from the activated fibroblasts further enhances BC cell migration by targeting E-cadherin, thereby contributing to in vivo tumor growth [160]. EV-lncRNA-CAF from oral squamous cell carcinoma cells activates CAF phenotype in NFs by stabling IL-33, thereby contributing to tumor proliferation in vitro and in vivo [161]. Additionally, MSCs serve as important recruited stromal cells in the TME and undergo reprogramming by tumors to harbor oncogenic potential. EV-miR-146a from multiple myeloma (MM) cells is transferred to MSCs and increases the expression and secretion of cytokines, including IL-6, CXCL1, IP-10, and CCL5 by activating Notch pathway, which further promotes the viability and migration of MM cells [162]. EV-miR-1587 from glioma-associated MSCs promotes the proliferation and clonogenicity of glioma stem-like cells by targeting NCOR1 [163]. Apart from the abundant tumor-promoting EV-RNAs within the TME, the reduction of tumor-suppressive EV-RNAs from cancer-educated noncancerous cells is also a general phenomenon contributing to tumorigenesis. EV-miR-320a from CAFs is reduced compared with NFs, which promotes the proliferation, migration and invasion of HCC cells by targeting PBX3 in vitro and in vivo [164]. EV-miR-3188 from CAFs is decreased compared with NFs, which enhances the proliferation and survival of head and neck cancer cells by targeting BCL2 in vitro and in vivo [165]. EV-miR-148b from CAFs is decreased compared with NFs, which promotes the EMT, migration and invasion of endometrial cancer cell by targeting DNMT1 in vitro and in vivo [166]. EV-miR-15a from MM BMSCs is reduced compared with normal BMSCs, which promotes MM cell proliferation [167].

In addition to CAFs and MSCs, other noncancerous cells are influenced by tumor-derived EV-RNAs to disrupt their normal phenotypes. EV-miR-155 from BC cells could promote brown differentiation and catabolism of adipocytes by targeting PPARγ [168]. Tumor-derived EV-miR-21 promotes apoptosis of skeletal muscle cells by activating TLR7 [169]. EV-ciRS-133 from gastric cancer cells promotes brown differentiation of preadipocytes and metabolic activity of adipocytes by targeting miR-133, thereby contributing to tumor cachexia in tumor-implanted mice [170]. In addition to cancer cachexia, EV-RNAs from cancer cells modulate bone remodeling and lesions. EV-lncRNA-RUNX2-AS1 from MM cells decreases osteogenic differentiation of MSCs by blocking RUNX2 splicing and could lead to osteolytic lesions in the bone marrow microenvironment [171]. EV-miR-940 from cancer cells induces the osteogenic differentiation of MSCs by targeting ARHGAP1 and FAM134A, thus triggering in vivo osteoblastic lesions in the bone metastatic microenvironment [172].

Tumor-promoting RNAs also disrupt vascular endothelial barriers and transform normal cells into niche cells in distant tissues and organs, leading to premetastatic niche generation and metastasis. For example, EV-miR-105 from BC cells damages vascular integrity by targeting tight junction protein ZO-1, resulting in enhanced vascular permeability and increased metastases in lung and brain [173]. EV-miR-103 from HCC cells promotes endothelial permeability and transendothelial invasion by targeting VE-Cadherin, p120 and ZO-1, thereby contributing to vascular permeability in tumor, cancer cell dissemination into the circulation and metastases in liver and lung [174]. EV-circRNA-IARS from pancreatic cancer cells promotes permeability of endothelial monolayers and transendothelial passage of cancer cells by targeting miR-122 [175]. Moreover, EV-miR-122 from BC cells is delivered to premetastatic niches and inhibits glucose consumption of lung fibroblasts and brain astrocytes by targeting PKM, thereby contributing to metastasis in the brain and lung [176]. EV-miR-1247-3p from highly metastatic HCC cells markedly improves the conversion of fibroblasts to CAFs by directly targeting B4GALT3 in lung premetastatic niches. Activated CAFs in turn secrete IL-6 and IL-8, pro-inflammatory cytokines, to promote cancer progression [177]. Apart from cancer-derived EV-RNAs modulating metastasis, EV-RNAs from normal cells also prime organs for metastasis. EV-miR-19 from astrocytes inhibits PTEN expression in metastatic tumor cells, and the tumor cells with PTEN loss recruit IBA1^+^ myeloid cells to increase proliferation and suppress apoptosis of themselves by secreting chemokine CCL2, thereby promoting brain metastasis in vivo [178]. EV-miR-92a from CD11b^+^ populations of bone-marrow derived cells (BMDCs) induces collagen type I expression and activation of hepatic stellate cells (HSCs) as well as lung cancer cell attachment on HSCs by targeting SMAD7, potentially leading to recruitment of granulocytic myeloid-derived suppressor cells (MDSCs), a immunosuppressive cell type, and liver metastasis in vivo [179].

During tumor development, malignant cancer cells or cancer stem cells can transfer oncogenic RNAs to less malignant cancer cells or noncancerous cells via EVs, thereby driving tumor growth and progression. The transferring of EV-RNAs from tumor to normal cells can trigger oncogenic transformation and inflammation. For example, cancer-derived EVs containing RISC-associated miRNAs induce oncogenic conversion of epithelial cells in a Dicer-dependent manner [180]. EV-miR-146b-5p from chronic myelogenous leukemia cells enhances oncogenic transformation of mononuclear cells into leukemia-like cells and genomic instability probably by targeting NUMB and BRCA1 [181]. NF-kB-mediated EV-miR-155 from arsenite-transformed hepatic epithelial cells induces pro-inflammatory phenotype of normal liver cells by activating IL-8, IL-6/STAT3 signaling [182]. EV-circRNA-100,284 from arsenite-transformed cells promotes the cell cycle and proliferation of normal hepatic cells by targeting miR-217, resulting in malignant transformation of the non-transformed cells [183]. The transferring of EV-RNAs within tumor disseminates malignant phenotypes between heterogeneous populations of cancer cells, including cancer stem cells and those varied in malignant degree. EV-miR-200 from highly metastatic BC cells transfers metastatic ability to neighboring or distant weakly metastatic BC cells, thereby promoting EMT and their colonization in lung [184]. EV-lncRNA-Sox2ot from highly invasive pancreatic ductal adenocarcinoma cells increases EMT and stemness of weakly invasive recipient cells by targeting miR-200c and upregulating Sox2, resulting in in vivo tumor metastasis [185]. EV-circRNA-PTGR1 from highly metastatic HCC cells confers metastatic potential to poorly metastatic HCC cells and promotes in vivo metastasis by targeting miR-449a [186]. Furthermore, EV-miR-146a-5p from colorectal cancer stem cells enhances stemness and sphere formation of recipient colorectal cancer cells by targeting Numb, leading to tumor growth in vivo [187]. EV-lncRNA-FMR1-AS1 from esophageal carcinoma stem cells could stimulate stemness of recipient cancer cells and in vivo tumor growth by binding TLR7 [188]. Interestingly, hypoxia, a hallmark of solid tumors, is an important external stimulus for dissemination of malignant behaviors between tumor cells. EV-miR-21 from hypoxic oral squamous cell carcinoma cells confers premetastatic behaviors to normoxic recipient cells, leading to tumor growth and metastasis in a xenograft model [189]. EV-lncRNA-UCA1 from hypoxic bladder cancer cells promotes the proliferation, migration and invasion of normoxic bladder cancer cells and bladder tumor growth in vivo [190].

During cancer treatment, therapy resistance of cancer cells is mediated by EV-RNAs from educated stromal cells and malignant cancer cells. EV-miR-21 from cancer-associated adipocytes and CAFs confers paclitaxel resistance to ovarian cancer cells and inhibits their apoptosis in vitro and in vivo by targeting APAF1 [191]. EV-miR-196a from CAFs reduces cisplatin sensitivity and apoptosis of head and neck cancer cells and promotes their proliferation in vitro and in vivo by targeting CDKN1B and ING5 [192]. Upon interaction with specific BC cells, EV-RN7SL1 from activated fibroblasts is devoid of SRP9/14 shielding to activate RIG-I in the BC cells, leading to inflammation, tumor growth, metastasis, and therapy resistance [193]. EV-lncRNA-H19 from CAFs confers stemness and oxaliplatin resistance to colorectal cancer cells by targeting miR-141, resulting in tumor growth and chemoresistance in vivo [194]. Moreover, EV-miR-222/223 from BC cell-primed BMSCs promotes the acquisition of quiescent and drug resistance phenotypes in BC cells; administration of anti-miR-222/223 transfected MSCs increases carboplatin efficiency and survival in a mouse model of dormant BC [195]. In addition to non-immune stromal cells, immune cells also secrete EV-RNAs and facilitate acquisition of therapy resistance. EV-miR-21 from M2 macrophages confers cisplatin resistance to gastric cancer cells and inhibits their apoptosis in vitro and in vivo by targeting PTEN [196]. EV-miR-365 from M2 macrophages confers gemcitabine resistance to pancreatic ductal adenocarcinoma cells by mediating pyrimidine metabolism, leading to chemotherapy resistance and shorter survival of tumor-bearing mice [197]. EV-lncRNA-HISLA from tumor-associated macrophages, a protumoral polarized cell type, promotes the aerobic glycolysis and apoptosis resistance of BC cells by stabilizing HIF-1α, leading to tumor glycolysis and chemoresistance in vivo [198]. EV-miR-21 from neuroblastoma cells is transferred to monocytes, which can differentiate into macrophages, and upregulates miR-155 expression by binding TLR8; the educated monocytes in turn secrete EV-miR-155 to induce chemotherapy resistance by targeting TERF1 in neuroblastoma cells [199]. EV-miR-126a from doxorubicin-induced MDSCs enhances induction of IL-13^+^ Th2 cells and tumor angiogenesis; systemic administration of miR-126a inhibitor and doxorubicin alleviates lung metastasis during breast tumor development [200]. Of note, cancer cells that undergo chemotherapy stress or become chemo-resistant disseminate therapy resistance via EV-RNAs within individual tumors. EV-miR-9-5p, miR-203a-3p, and miR-195-5p from chemo-stressed BC cells could confer stemness and docetaxel resistance to recipient BC cells by jointly targeting ONECUT2 [201]. EV-lncRNA-VLDLR from chemo-stressed HCC cells confers chemoresistance to recipient cancer cells by upregulating ABCG2 expression [202]. EV-miR-151a from temozolomide-resistant glioblastoma multiforme cells confers chemoresistance to recipient sensitive cancer cells in vitro and in vivo by targeting XRCC4 [203]. EV-miR-222-3p from gemcitabine-resistant lung cancer cells promotes the growth, migration, invasion, gemcitabine resistance and anti-anoikis of recipient sensitive cancer cells by targeting SOCS3, leading to lung and other organ metastasis [204]. EV-lncRNA-ARSR from resistant renal cell carcinoma cells disseminates sunitinib resistance to recipient sensitive cancer cells in vitro and in vivo by targeting miR-34 and miR-44 9[205].

In addition, cancer cells also discard tumor-suppressive miRNAs to maintain and promote their oncogenic ability via EVs. miR-23b expression is upregulated in EVs of metastatic bladder cancer cells than nonmetastatic cells and reduced exocytosis of miR-23b via Rab27b knockdown promotes its intracellular activity; miR-23b inhibits invasion and anoikis of metastatic bladder cancer cells, thereby reducing in vivo angiogenesis and lung colonization [206]. miR-6126 expression is upregulated in EVs of ovarian cancer cells than that in their secreted cells or normal ovarian epithelial cells, and miR-6126 inhibits migration, invasion of ovarian cancer cells and tumor growth in vivo by targeting integrin-b1 [207]. miR-940 expression is upregulated in EVs of ovarian cancer cells than that in their secreted cells or normal ovarian cells, and miR-940 inhibits the proliferation, invasion, and migration of ovarian cancer cells and tumor growth in vivo as well as triggers their apoptosis by targeting SR C[208].

### Regulation of tumor-promoting functions of endothelial cells by tumor EV-RNAs

EV-RNAs of cancer cells enhance the proliferation, migration and tube formation of endothelial cells, thereby contributing to tumor and lymphatic vasculature. EV-miR-210 from HCC cells promotes tube formation of human umbilical vein endothelial cells (HUVECs) by targeting SMAD4 and STAT6, leading to in vivo angiogenesis and tumor growth [209]. EV-piRNA-823 from MM cells decreases the apoptosis and increases the proliferation, invasion and tube formation of HUVECs, leading to in vivo angiogenesis and tumor growth [210]. EV-miR-26a from glioma stem cells promotes the proliferation, migration and tube formation of human brain microvascular endothelial cells (HBMECs) by targeting PTEN [211]. EV-lncRNA-H19 from CD90^+^ liver cancer cells promotes tube formation and adhesive ability of HUVECs [212]. EV-RNA-mediated angiogenesis, lymphangiogenesis and vascular permeability can facilitate cancer cell dissemination and prime premetastatic niche formation. For example, EV-miR-23a, which is associated with metastasis, from nasopharyngeal carcinoma cells promotes the growth, migration and tube formation of HUVECs by repressing TSGA10, thereby contributing to in vivo angiogenesis [213]. EV-miR-221-3p from cervical squamous cell carcinoma enhances the migration and tube formation of lymphatic endothelial cells by directly targeting VASH1, leading to lymphangiogenesis and lymphatic metastasis in vivo [214]. EV-miR-25-3p from colorectal cancer stimulates angiogenesis and vascular permeability by targeting KLF2 and KLF4, thereby contributing to premetastatic niche formation and metastasis in the liver and lung [215]. As a driving force for angiogenesis, hypoxia stimulates the secretion of pro-angiogenic EV-RNAs from cancer cells and contributes to cancer progression. For example, EV-miR-135b from hypoxic MM cells promotes tube formation of normoxic and hypoxic HUVECs and in vivo neovascularization by targeting FIH-1 [216]. EV-miR-23a from hypoxic lung cancer cells increases tube formation, permeability and cancer cell transendothelial invasion of normoxic and hypoxic HUVECs by targeting PHD1, PHD2 and ZO-1, thereby promoting neovascularization and tumor growth in vivo [217]. Moreover, cancer-derived EV-RNAs also trigger proangiogenic shift of CAFs to induce tumor angiogenesis. EV-miR-155-5p from melanoma cells induces CAF phenotype and enhances the secretion of proangiogenic factors, including VEGFa, FGF2, MMP9, in fibroblasts by targeting SOCS1, leading to angiogenesis in vitro and in vivo [218]. EV-miR-21 from HCC cells triggers conversion of normal HSCs into CAFs by targeting PTEN, and activated CAFs in turn promote angiogenesis in vitro and in vivo by secreting angiogenic cytokines, including TGF-β, VEGF, bFGF, MMP2 and MMP9 [219]. Notably, EV-RNAs from noncancerous cells can exert anti-tumor effects by inhibiting tumor angiogenesis. EV-miR-15a, miR-181b, miR-320c and miR-874 from liver stem-like cells inhibit the migration and tube formation of tumor-derived endothelial cells and potential tumor angiogenesis by downregulating FGF1 and PLAU [220]. EV-miR-100 from MSCs inhibits the expression and secretion of VEGF in BC cells by targeting mTOR, which decreases the proliferation, migration and tube formation of HUVEC [221]. Interestingly, cancer cells could decrease tumor-suppressive EV-RNA secretion to remove restraint in tumor growth. EV-miR-9 from nasopharyngeal carcinoma cells, the expression of which is reduced than that of normal nasopharyngeal cells, suppresses the migration and tube formation of HUVECs and angiogenesis in vivo by targeting MDK [222].

### Regulation of cancer immunology and inflammation by tumor and immune EV-RNAs

EV-RNAs of cancer cells have been shown to modulate the functions and cytokine secretion of immune cells, thereby regulating anti-tumor immune response and immune evasion **(**Fig. 4**)**. Macrophages have proinflammatory M1 and anti-inflammatory M2 polarizations in immunity. Tumor-associated macrophages, primary infiltrative immune cells of the TME, are generally classified into M1-like anti-tumoral and M2-like protumoral phenotypes [223]. The pro-cancerous crosstalk mediated by EV-RNAs is also available for macrophages, leading to the M2-like phenotype shift and then cancer progression. For example, EV-miR-1246 from colon cancer cells with mutant p53 educates macrophages into tumor-promoting phenotype [224]. EV-miR-21 from head and neck cancer cells overexpressing snail triggers M2 macrophage polarization by downregulating PDCD4 and IL12A [225]. EV-lncRNA-RPPH1 from colorectal cancer cells promotes macrophage M2 polarization, resulting in tumor growth and metastasis [226]. Furthermore, M2 macrophage-derived EV-RNAs also participate in cancer progression. EV-miR-21-5p and miR-155-5p from M2 macrophages induce the migration and invasion of colorectal cancer cells by jointly targeting BRG1, leading to in vivo lung metastasis [227]. EV-miR-501-3p from M2 macrophages enhances the migration, invasion and tube formation of pancreatic ductal adenocarcinoma cells by targeting TGFBR3, resulting in tumor growth and metastasis in the lung and liver [228]. Importantly, hypoxia contributes to active communication between cancer cells and immune cells, such as macrophages, T cells, MDSCs and natural killer (NK) cells, serving as a driving force for immunosuppressive microenvironment and cancer progression. Taking macrophages as the first example, hypoxic conditions increase tumor-derived EV-RNA secretion and corresponding M2 polarization effects on macrophages, and M2 macrophages increase the malignant potential of cancers by secreting cytokines. EV-miR-301a from hypoxic pancreatic cancer cells induces the M2 polarization of macrophages by targeting PTEN, which in turn promotes the migration, invasion, EMT and lung metastasis of pancreatic cancer cells probably by secreting IL10, TGF-β and arginase-1 [229]. EV-miR-103a from hypoxic lung cancer cells enhances M2 macrophage polarization by targeting PTEN, which further promotes the migration and invasion of lung cancer cells and tube formation of HUVECs by secreting IL-10, CCL18 and VEGF- A[230]. EV-miR-1246 from hypoxic glioma cells reprograms macrophages into M2 phenotype by targeting TERF2IP, which further enhances the proliferation, migration and invasion of glioma cells in vitro and in vivo probably by secreting IL10, TGF-β [231].

EV-RNA-mediated immune reprogramming also impedes T-cells function in direct and indirect manners, thus promoting the immune escape of cancer cells. In an direct manner, EV-miR-29a-3p and miR-21-5p from M2 macrophages increase regulatory T cell (Treg)/Th17 ratio by jointly targeting STAT3, which could promote tumor growth and metastasis and reduce survival time in a ovarian cancer mouse model [232]. EV-miR-214 from tumor cells triggers IL-10 secretion and expansion of Tregs, immunosuppressive T cells, by targeting PTEN, thereby facilitating immunosuppression and tumor growth in vivo [233]. EV-miR-24-3p from nasopharyngeal carcinoma cells induces differentiation of Tregs and inhibits T-cell proliferation as well as Th1 and Th17 differentiation by targeting FGF11 [234]. Under hypoxic condition, the above immunosuppressive effects of EVs are enhanced due to the increased secretion of EV-miR-24-3p. EV-RNAs from head and neck cancer cells trigger an immunosuppressive phenotype in CD8^+^ T cells, which inhibits the proliferation of nearby normal T cells [235]. EV-circRNA-002178 from lung cancer cells enhances the PD-1 expression of CD8^+^ T cells by targeting miR-28-5p, thereby potentially triggering T-cell exhaustion [236]. Alternatively, recent research focusing on T cell dysfunction demonstrated cancer-derived EV-RNAs facilitate macrophage polarization and MDSC infiltration in the TME, which in return exerts inhibitory activities on effector T cells. Taking macrophage polarization as the first example, EV-miR-23a-3p from endoplasmic reticulum-stressed HCC cells promotes the expression of PD-L1 in macrophages by targeting PTEN, which could reduce CD8^+^ T-cell proportion and induce apoptosis in T cells [237]. EV-miR-503 from XIST-knockdown BC cells induces the M2 phenotype and PD-L1 expression of microglia probably by mediating STAT3 and NF-κB pathways, which suppresses the proliferation of T-cells [238]. EV-miR-146a-5p from HCC cells induces M2 polarization of macrophages and could enhance T-cell exhaustion, which is mediated by transcription factor SALL4 during HCC development [239]. As for MDSC infiltration, EV-miRNAs from melanoma cells induce the conversion of monocytes into MDSCs and then suppress T cell activation and cytokine secretion [240]. EV-miR-21 from hypoxic oral squamous cell carcinoma cells inhibits the cytotoxicity and proliferation of γδ T-cells by promoting MDSC expansion and regulating PTEN/PD-L1 axis; combination of anti-PD-L1 treatment and miR-21 knockdown attenuates the protumoral effects of tumor-derived EVs in vivo [241]. EV-miR-10a and miR-21 from hypoxic glioma cells promote MDSC propagation and their immunosuppressive abilities on CD8^+^ T cells by targeting RORA and PTEN, respectively [242]. EV-miR-29a and miR-92a from hypoxic glioma cells stimulate MDSC differentiation and their immunosuppressive activities on CD8^+^ T cells by targeting Hbp1 and Prkar1a, respectively [243]. In addition, other immune cells are reprogramed by tumor-derived EV-RNAs and participate in immunosuppression in the TME. EV-miR-212-3p from pancreatic cancer cells decreases MHC II transcription factor RFXAP expression of dendritic cells by targeting RFXAP, leading to reduced MHC II expression and potential immune tolerance [244]. EV-miR-203 from pancreatic cancer cells reduces TLR4 expression and the release of cytokines, including TNF-a and IL-12, in dendritic cells [245]. EV-Y RNA-hY4 from chronic lymphocytic leukemia cells triggers PD-L1 expression and cytokine secretion in monocytes via TLR7 signaling, leading to pro-tumorigenic inflammation and potential immune escape [246]. EV-miR-23a from hypoxic tumor cells reduces the cytotoxicity of NK cells by targeting CD107a [247]. 5-phosphates exosomal RNAs from colorectal cancer stem cells induce the IL-1β expression of neutrophils probably by activating RIG-I, thereby promoting survival and expansion of neutrophils for tumor infiltration [248]. Of note, EV-RNAs from immune cells also exert anti-tumor effect on cancer cells. EV-miR-7 from TWEAK-stimulated macrophages inhibits the migration and invasion of epithelial ovarian cancer cells by targeting EGFR [249]. EV-miR-186 from NK cells induces cytotoxicity to MYCN-amplified neuroblastoma cells; targeted delivery of miR-186 suppresses tumor growth and improves the survival of a orthotopic mouse model of neuroblastoma by targeting MYCN, AURKA, TGFΒR1 and TGFΒR2 [250].

EV-RNAs from cancer cells can serve as damage-associated molecular patterns (DAMPs) to activate pattern recognition receptors (PRRs) and trigger inflammatory response and premetastatic niche formation, thereby driving cancer progression. EV-miR-21 and miR-29a from lung cancer cells induce NF-κB activation and the release of prometastatic inflammatory cytokines in murine and human macrophages by activating TLR7 and TLR8 respectively, which promotes lung cancer metastasis [251]. Tumor-derived EVs containing small nuclear RNAs induce chemokine production of alveolar epithelial cells by activating TLR3 and subsequent lung neutrophil infiltration, thereby contributing to premetastatic niche formation and metastasis in the lung [252]. EV-miR-21 from colorectal cancer cells polarizes macrophages to secrete IL-6 by activating TLR7, leading to inflammatory premetastatic niche and metastasis in the liver [253].

## Conclusions

Regulation of cancer cells and the TME by EV-RNAs has been shown to be an important aspect in tumorigenesis. The main interest in this field has focused on biological roles of tumor-promoting EV-RNAs, especially miRNAs, within the TME. Tumor-promoting EV-RNAs are involved in the cancer cell-stromal cell, immune cell-immune cell and cancer cell-cancer cell crosstalks, thereby promoting the initiation, growth, angiogenesis and survival of primary tumor as well as multiple steps of the metastatic process, including local invasion, intravasation, extravasation and outgrowth of cancer cells at metastatic sites. Premetastatic niche formation mediated by tumor- or normal cell-derived EV-RNAs is characterized by vascular permeability, angiogenesis, metabolic reprogramming, ECM remodeling, immunosuppression and inflammatory microenvironment. Hypoxia is considered as a major driving force for shaping the TME and induces most, if not all, of cancer malignant phenotypes by increasing tumor-promoting EV-RNA release. Hypoxia-induced cancer progression mediated by EV-RNAs is attributed to protumoral niches fostered by normal cells, endothelial permeability, angiogenesis, malignant evolution within tumors and immunosuppressive microenvironment. Interestingly, tumor-promoting EV-miR-21 promotes angiogenesis, immunosuppression and the malignancy of many cancers by involving in complex communication networks. As for tumor-suppressive RNAs, there is now a focus on their safe and effective delivery to cancer cells by manipulating donor cells or directly loading into EVs. Of note, the downregulation of tumor-suppressive EV-RNAs in the TME may be a general phenomenon and require further investigation. Moreover, EV-RNA-mediated immune dysfunction and inflammation in cancers are emerging topics in research fields, and EV-RNAs regulate both innate and adaptive immune systems of the host by participating in cancer cell-immune cell and immune cell-immune cell crosstalks. The tumor-derived EV-RNAs contribute to not only decreased anti-tumor response of T cells and NK cells but also induction of immunosuppressive cells, such as CAFs, tumor-associated macrophages, MDSCs and Tregs, which further restricts tumor-suppressive functions of CD8^+^ T cells. Pro-inflammatory and anti-inflammatory effects of EV-RNAs are associated with cancer progression. As key mediators of inflammatory conditions in cancers, macrophages foster protumoral anti-inflammatory and prometastatic inflammatory microenvironment upon receiving tumor-derived EV-RNAs.

Despite growing studies regarding miRNAs, lncRNAs and emerging circRNAs affecting cancer hallmarks, the in vivo roles and addressability of EV-RNAs in cancer biology remain largely unknown. Xenograft mouse models have been commonly used in EV-RNA studies where EVs isolated from cell lines are injected into tumor or blood circulation. This approach can not reflect the spatiotemporal properties, concentration and targeting of EV-RNAs in pathological processes. Moreover, stoichiometric analysis of EV-RNAs indicated that single exosome contains far less than one copy of miRNAs on average, and majority of individual exosomes does not have functional numbers of miRNAs [254]. In this context, the EVs concentration is an important factor for the dose-dependent effect of RNAs on target cells. It is hypothesized that diverse RNAs could work together to simultaneously mediate cancer hallmarks by jointly regulating a single pathway or mRNAs [255]. Therefore, corporation between EV-RNAs may be another important factor for augmenting their ability affecting tumorigenesis. The complementary roles of EV-RNAs in cancer biology remain largely unexplored and require further research. Of note, most published papers of EV-RNAs have focused on RNA content and functions of exosomes rather than microvesicles in cancers. Different types of EVs possess distinct biological properties and RNA content, which could affect their distribution and functions in the TME. Therefore, characterization of RNA content, delivery and functions of different EV subpopulations contributes to expand our knowledge of EV-RNA-mediated cell-cell communication in cancer biology.

## Data Availability

Not applicable.

## Abbreviations

AMSCs: Adipose tissue-derived mesenchymal stem cells
ARF6: ADP ribosylation factor 6
ARRDC1: Arrestin-domain-containing protein 1
BC: Breast cancer
BMSCs: Bone marrow mesenchymal stem cells
CAFs: Cancer-associated fibroblasts
CAV-1: Caveolin-1
circRNAs: Circular RNAs
DAG: Diacylglycerol
DAMPs: Damage-associated molecular patterns
ECM: Extracellular matrix
EMT: Mesenchymal-to-epithelial transition
ERK: Extracellular signal-regulated kinase
ESCRT: Endosomal sorting complex required for transport
EVs: Extracellular vesicles
HAS3: Hyaluronan synthase 3
HCC: Hepatocellular carcinoma
HSCs: Hepatic stellate cells
HUVECs: Human umbilical vein endothelial cells
ILVs: Intraluminal vesicles
IS: Immune synapse
LIMK: Lim kinase
lncRNAs: Long non-coding RNAs
MDSCs: Myeloid-derived suppressor cells
miRISCs: miRNA-loaded RNA-induced silencing complexs
miRNAs: Micro RNAs
MLC: Myosin light-chain
MLCK: Myosin light-chain kinase
MLCP: Myosin light chain phosphatase
MM: Multiple myeloma
mRNAs: Messenger RNAs
MSCs: Mesenchymal stem cells
MTOC: Microtubule-organizing center
MVEs: Multivesicular endosomes
ncRNAs: Non-coding RNAs
NFs: normal fibroblasts
NK: Natural killer
PADs: Peptidylarginine deiminases
PAR2: Protease activated receptor 2
PLD: Phospholipase D
PRRs: Pattern recognition receptors
RBPs: RNA binding proteins
ROCK: RHO-associated protein kinase
S1P: Sphingosine 1-phosphate
SNARE: Soluble N-ethylmaleimide-sensitive factor attachment protein receptor
TME: Tumor microenvironment
Treg: Regulatory T cell
t-SNAREs: Target-membrane SNAREs
v-SNAREs: Vesicle-membrane SNAREs
